# Rapid Onset of Fulminant Type 1 Diabetes 10 Days After the First Dose of Nivolumab: A Case Report

**DOI:** 10.7759/cureus.85957

**Published:** 2025-06-13

**Authors:** Yoshinori Miyazaki, Tomomi Tateyama, Haruo Shimizu

**Affiliations:** 1 Diabetology, Muroran City General Hospital, Muroran, JPN; 2 Gastroenterology, Muroran City General Hospital, Muroran, JPN

**Keywords:** fulminant type 1 diabetes, gastric cancer, immune-related adverse events, nivolumab, ten days after the initial treatment

## Abstract

A 70-year-old man was admitted to our hospital for eight days and received treatment with nivolumab, a programmed cell death (PD)-1 inhibitor, for gastric cancer with liver metastasis. On the 10th day after the first administration of nivolumab, he developed symptoms of appetite loss, general malaise, and consciousness disturbance, and he was readmitted to our hospital. Laboratory tests showed ketonuria, metabolic acidosis (pH 6.978; bicarbonate ion level of 1.7 mmol/L), and hyperglycemia (glucose level of 992 mg/dL). His hemoglobin A1c (HbA1c) level was slightly elevated (7.3%), while his HbA1c levels 9 years, 33 days, and 22 days before the administration of nivolumab were 6.0%, 6.1%, and 6.1%, respectively. His serum C-peptide level and 24-hour urinary total C-peptide levels were less than the detection limits. His serum amylase and lipase levels were elevated to more than three times the upper reference limits. He tested negative for islet autoantibodies, including anti-glutamic acid decarboxylase (GAD), anti-insulinoma-associated protein 2 (IA-2), and anti-zinc transporter 8 (ZnT8). The findings in this case indicated that nivolumab caused new-onset fulminant type 1 diabetes (FD). To the best of our knowledge, this case represents the shortest reported time to the development of FD following the first injection of a PD-1 inhibitor. Attention should be paid to the elevation of glucose level even in the very early phase after initial administration of nivolumab.

## Introduction

Immune checkpoint inhibitors (ICIs), such as programmed cell death/programmed death ligand 1 (PD-1/PD-L1) inhibitors and T-lymphocyte-associated protein 4 (CTLA-4) inhibitors, have been widely used as a standard cancer treatment in recent years. However, occasionally, ICIs cause immune-related adverse events (irAEs), affecting endocrine glands in up to 40% of patients [1], depending on the drugs used. The most common endocrine organs affected by ICIs are the thyroid and pituitary glands, affected in up to 20% and 10% of cases, respectively [1]. The occurrence of ICI-associated diabetes as type 1 diabetes is relatively rare, with an estimated incidence of around 1% [1-3], but it is potentially life-threatening irAE, and evidence regarding clinical course and pathogenesis of ICI-associated type 1 diabetes remains limited. Furthermore, the time from the initial ICI administration to diabetes onset in previously reported cases varied from days to years, and the mean or median period was from 49 to 183 days according to a large clinical study or systematic review [2-4]. We herein report a patient with gastric cancer and liver metastasis who developed fulminant type 1 diabetes (FD) on the 10th day after the first administration of nivolumab, a PD-1 inhibitor. To the best of our knowledge, the period from the first ICI administration to the onset of FD in our case is the shortest period so far reported.

## Case presentation

A 70-year-old male was diagnosed with gastric cancer with liver metastasis and was hospitalized one month later in the Department of Gastroenterology at our hospital. On the next day after hospitalization (day 0), treatment with nivolumab (360 mg once on day 0) and S-1 (100 mg on days 0-13) plus oxaliplatin (245 mg once on day 0) was started. Steroids were not used for the treatment. During hospitalization for eight days, no obvious adverse reactions were noted, and the patient had no upper respiratory or gastrointestinal symptoms. Although laboratory data were almost stable during the hospitalization, slight decreases in concentrations of sodium and chloride on the date of discharge (day 6) (Table 1) were observed, suggesting that plasma glucose might have begun to increase rapidly within 10 days after the first nivolumab injection. To our regret, the gastroenterologists, who were in charge of the treatment, did not measure glucose levels during the hospitalization between days -1 and 6. On the 10th day (day 10) after the first injection, four days after discharge from the hospital, he was brought to the emergency room of our hospital with symptoms of appetite loss, general malaise, tachycardia, and consciousness disorder. On physical examination, the patient’s blood pressure was 88/59 mmHg, his pulse rate was 109 beats/minute, and his body temperature was 35.0 °C. His height and weight were 173 cm and 67 kg, respectively. There was no abdominal tenderness. Laboratory tests (Table 1) showed ketonuria, metabolic acidosis (pH 6.978; bicarbonate ion level of 1.7 mmol/L), and hyperglycemia (glucose level of 992 mg/dL), and he was immediately hospitalized again. His hemoglobin A1c (HbA1c) level was slightly elevated (7.3%). His serum C-peptide level and 24-hour urinary total C-peptide level were less than the detection limits. His serum amylase and lipase levels were elevated to more than three times the upper reference limits, although a non-contrast on day 10 and a contrast-enhanced abdominal computed tomography on day 12 did not show signs of pancreatitis. He was negative for islet autoantibodies (anti-GAD antibody, anti-IA2 antibody, and anti-ZnT8 antibody). He had no personal or family history of diabetes, and his HbA1c levels at 9 years, 33 days, and 22 days before the first administration of the nivolumab were 6.0%, 6.1%, and 6.1%, respectively. He had a 20-year history of gastric carcinoid and chronic thyroiditis, a 12-year history of hypertension, and a 9-year history of atherothrombotic cerebral infarction treated with rabeprazole at 10 mg/day, levothyroxine at 50 mg/day, bisoprolol at 2.5 mg/day, irbesartan at 100 mg/day, amlodipine at 5 mg/day, indapamide at 1 mg/day, and clopidogrel at 75 mg/day. Human leukocyte antigen (HLA) typing showed haplotype DRB1^*^09:01-DQB1^*^03:03, which is a haplotype strongly associated with autoimmune acute-onset type 1 diabetes in Japan [5]. The findings in this case indicated that nivolumab caused new-onset FD. The patient initially received isotonic saline and continuous insulin infusion. On the third hospital day, upon resolution of diabetic ketoacidosis, he was switched from continuous insulin infusion to multiple daily subcutaneous injections of insulin. At discharge, 20 days after the onset of FD (on day 30), his insulin regimen included 12 units of basal and 32 units of bolus insulin. Free thyroxine (FT4) was within normal range (1.14 ng/dL), and thyroid-stimulating hormone (TSH) was slightly elevated (5.84 µIU/mL) during levothyroxine treatment (50 µg/day), which had started 22 days before the first nivolumab administration. However, after admission (on day 13), FT4 was elevated (2.35 ng/dL) and TSH was suppressed (0.06 µIU/mL), leading to the discontinuation of levothyroxine. However, the patient developed hypothyroidism (FT4 0.80 ng/dL; TSH 6.20 µIU) again during admission (on day 29), and we resumed treatment with levothyroxine at 50 mg/day and increased the dose to 150 mg/day for recovery of thyroid function.

**Table 1:** Laboratory findings of the patient before and after the nivolumab treatment on day 0. N/R, not reported; WBC, white blood cell count; RBC, red blood cell count; Hb, hemoglobin; Ht, hematocrit; Plt, Platelet; TP, total protein; Alb, albumin; AST, aspartate aminotransferase; ALT, alanine aminotransferase; LDH, lactate dehydrogenase; ALP, alkaline phosphatase; γGTP, γ-glutamyl transpeptidase; T-Chol, total cholesterol; TG, triglyceride; Cre, creatinine; GFR, estimated glomerular filtration rate; BUN, blood urea nitrogen; Na, sodium; K, potassium; Cl, chloride; UACR, urinary albumin-to-creatinine ratio; FT4, free thyroxine; TSH, thyroid-stimulating hormone; U-Protein, urinary protein; U-Glucose, urinary glucose; U-Ketone, urinary ketone; HCO3-, bicarbonate ion; F-CPR, fasting c-peptide immunoreactivity; U-CPR, urinary c-peptide immunoreactivity; Ins Ab, anti-insulin antibody; GAD Ab, anti-glutamic acid decarboxylase antibody; IA2 Ab, anti-islet antigen 2 antibody; ZnT8 Ab, anti-zinc transporter isoform 8 antibody; Tg Ab, thyroglobulin antibody; TPO Ab, thyroid peroxidase antibody; TSHR Ab, thyroid stimulating hormone receptor antibody; ACTH, adrenocorticotropic hormone

	Day -33	Day -22	Day -1	Day 2	Day 6	Day 10	Day 13	Reference range
WBC	6,540	6,330	7,610	8,410	8,090	31,550	10,750	3,900-9,800/µL
RBC	435	407	411	442	458	374	401	425-570x10^4^/µL
Hb	13.4	12.5	12.6	13.7	13.9	11.5	12.4	13.5-17.6 g/dL
Ht	39.8	37.1	37.3	39.7	39.7	31.7	35.1	40-52%
Plt	19.2	24.8	20.7	22.7	19.9	32.1	12.4	15-40x10^4^/µL
TP	6.9	6.8	6.7	6.8	6.6	6.4	5.5	6.5-8.0 g/dL
Alb	4.1	3.9	4.1	4.1	3.8	3.8	3.1	3.8-5.3 g/dL
AST	30	31	25	61	48	40	38	10-45 U/L
ALT	20	23	19	37	37	31	24	5-43 U/L
LDH	223	248	237	262	299	343	270	124-222 U/L
ALP	83	92	87	76	84	142	115	38-113 U/L
γGTP	101	101	82	79	90	76	64	16-73 U/L
Amylase	187	241	164	152	206	N/R	606	40-150 U/L
Lipase	N/R	N/R	254	195	N/R	N/R	2795	11-53 U/L
T-Chol	177	168	170	198	174	N/R	110	130-219 mg/dL
TG	122	N/R	152	151	111	N/R	93	50-149 mg/dL
Cre	1.18	1.14	1.10	1.18	1.14	2.26	0.91	0.2-1.0 mg/dL
eGFR	47.8	49.7	51.6	47.8	49.7	23.5	53.2	90< mL/minute
BUN	16.0	17.5	14.3	16.3	17.8	42.0	12.8	8-22 mg/dL
Na	136	136	136	137	126	119	127	135-148 mmol/L
K	3.9	4.4	3.7	3.7	3.8	6.1	4.4	3.5-5.0 mmol/L
Cl	97	99	98	98	88	81	90	98-108 mmol/L
Glucose	114	113	N/R	N/R	N/R	992	356	70-109 mg/dL
HbA1c	6.1	6.1	N/R	N/R	N/R	N/R	7.3	4.6-6.2%
UACR	N/R	N/R	N/R	N/R	N/R	N/R	58.9	<30 mg/gCr
FT4	N/R	1.14	N/R	N/R	N/R	N/R	2.35	0.70-1.48 ng/dL
TSH	N/R	5.84	N/R	N/R	N/R	N/R	0.06	0.38-5.38 µIU/mL
U-Protein	(-)	(-)	N/R	N/R	N/R	(+/-)	(-)	(-) or (+/-)
U-Glucose	(-)	(-)	N/R	N/R	N/R	4+	4+	(-) or (+/-)
U-Ketone	(-)	(-)	N/R	N/R	N/R	3+	2+	(-)
pH	N/R	N/R	N/R	N/R	N/R	6.978	N/R	7.35-7.46
HCO_3_^- ^	N/R	N/R	N/R	N/R	N/R	1.7	N/R	19-31 mmol/L
pCO_2_	N/R	N/R	N/R	N/R	N/R	7.8	N/R	35-45 mmHg
F-CPR	N/R	N/R	N/R	N/R	N/R	N/R	<0.03	0.61-2.09 ng/mL
U-CPR	N/R	N/R	N/R	N/R	N/R	N/R	<1.8	29-167 µg/day
Ins Ab	N/R	N/R	N/R	N/R	N/R	N/R	<0.4	<0.4 U/mL
GAD Ab	N/R	N/R	N/R	N/R	N/R	N/R	<5.0	<5.0 U/mL
IA2 Ab	N/R	N/R	N/R	N/R	N/R	N/R	<0.6	<0.6 U/mL
ZnT8 Ab	N/R	N/R	N/R	N/R	N/R	N/R	<10.0	<10.0 U/mL
Tg Ab	N/R	N/R	N/R	N/R	N/R	N/R	1730	<19.3 IU/mL
TPO Ab	N/R	N/R	N/R	N/R	N/R	N/R	1390	<3.3 IU/mL
TSHRs Ab	N/R	N/R	N/R	N/R	N/R	N/R	103	<121%
ACTH	N/R	13.1	N/R	N/R	N/R	N/R	13.6	7.2-63.3 pg/mL
Cortisol	N/R	7.4	N/R	N/R	N/R	N/R	12.5	4.0-18.3 µg/dL

Two months after the onset of FD (on day 69), nivolumab treatment was resumed (second dose), and the third dose was administered on day 90, alongside continued self-injected insulin therapy, without recurrence of FD. Multiple daily insulin injections remained necessary, and the insulin dosage was increased to maintain plasma glucose control. His HbA1c levels were 8.6% on day 58, 8.9% on day 114, and 7.2% on day 156 under treatment with multiple insulin injections. On day 156, his fasting C-peptide level (<0.03 ng/mL) and anti-GAD antibody (<5.0 U/mL) were still below the detection limits.

## Discussion

We present a case of new-onset FD 10 days after the initiation of nivolumab therapy for gastric cancer. FDis a distinct subtype of type 1 diabetes first identified in Japan. It is clinically characterized by a markedly rapid onset of hyperglycemia with ketoacidosis, a near-normal HbA1c level (<8.7%) despite severe hyperglycemia (≥288 mg/dL or 16.0 mmol/L), and either a urinary C-peptide level of <10 µg/day at onset or a fasting blood C-peptide level of <0.3 ng/mL [6]. Additional features of FD may include elevated serum pancreatic enzymes and, in principle, negative islet-related autoantibodies. In the present case, all conditions were met, and the patient was, therefore, diagnosed with FD. ICI-induced type 1 diabetes is relatively rare, with an estimated incidence of around 1% [1-3], but it is a potentially life-threatening irAE. Approximately 97% of all reported cases of ICI-induced diabetes occurred in patients treated with PD-1/PD-L1 inhibitor or a combination of PD-1/PD-L1 inhibitor plus CTLA-4 inhibitor, whereas reports of cases on CTLA-4 inhibitor monotherapy are very rare [3,7]. According to a safety database in Japan [4], nivolumab-related and pembrolizumab-related cases of type 1 diabetes account for 0.33% and 0.14% of all cases of irAEs, respectively, and 50.0% of the patients with PD-1 inhibitor-related type 1 diabetes fulfilled the criteria of FD. In addition, according to the database, the mean period from the first ICI injection to the onset of type 1 diabetes was 155 ± 123 days (range, 13-504 days) [4]. Furthermore, the mean or median period was from 49 to 183 days according to a large clinical study or systematic review [2-4]. Hughes et al. [8] reported a case of type 1 diabetes that developed seven days after the initial nivolumab administration, while the patient had preexisting type 2 diabetes treated with metformin, and the patient’s HbA1c level was 9.7%. Akturk et al. [9] reported a case of new-onset severe diabetic ketoacidosis that developed five days after the initial nivolumab administration, representing the shortest interval from first ICI exposure to the onset of type 1 diabetes among previously reported cases, based on our review. However, both reported cases [8,9] were positive for anti-GAD antibody at the time of diagnosis of nivolumab-induced type 1 diabetes, suggesting acute exacerbation of slowly progressive insulin-dependent (type 1) diabetes mellitus by nivolumab treatment. Furthermore, HbA1c and C-peptide levels at the time of diagnosis were not reported in the case described by Akturk et al. [9], making it unclear whether the diagnostic criteria for FD were met. To the best of our knowledge [2-4,8-10], the time to the development of FD after the first ICI injection in our case is the shortest period in cases of ICI-associated FD.

In the present case, results for pancreas autoantibodies (anti-GAD antibody, anti-IA2 antibody, anti-ZnT8 antibody, and anti-insulin antibody) were negative. According to the safety database of ICI-associated type 1 diabetes in Japan, only one of 22 patients was positive for anti-GAD antibody, and no patients were positive for any other antibodies [4]. Other systematic reviews for Western patients showed that more than 50% of patients with ICI-induced type 1 diabetes were positive for islet autoantibodies, the most common of which was anti-GAD antibody [2,7]. Gauci et al. also suggested that the presence of anti-GAD antibody is related to the shortness of the period from the initial ICI treatment to the onset of ICI-associated type 1 diabetes [10], but that possibility remains to be elucidated. In any case, the pathogenesis in ICI-associated type 1 diabetes seems to differ at least partly from that of conventional autoimmune type 1 diabetes involving islet autoantibodies. Considering that conventional FD develops mainly in East Asia and rarely in Western countries [11], the racial difference in the positive rate of anti-GAD antibody in patients with ICI-induced type 1 diabetes might be related to the difference in the human leucocyte antigen (HLA) [4].

The genotypic combination of HLA typing in our patient revealed DRB1^*^09:01-DQB1^*^03:03/DRB1^*^15:02-DQB1^*^06:01 without anti-GAD antibody. In a large-scale study in Japan, Tsutsumi et al. [12] re-evaluated the association of HLA genotype with classic FD in patients with or without anti-GAD antibody and reported that DRB1^*^09:01-DQB1^*^03:03 was strongly associated with classic FD in only homozygous state with or without anti-GAD antibody, while the haplotype in a heterozygous state was predominant in only antibody-positive FD patients, indicating that the HLA typing in our case does not relate to classic FD according to the report by Tsutsumi et al. In Caucasians, certain HLA typing has also been reported to predispose to type 1 diabetes, including DRB1^*^03:01-DQB1^*^02:01 and DRB1^*^04:01-DQB1^*^03:02 [13]. Based on clinical case reports and a review of the literature regarding ICI-induced type 1 diabetes [14,15], patients with high-risk HLA for type 1 diabetes may have an increased risk for the development of ICI-induced type 1 diabetes. However, the clinical features of and data regarding ICI-induced type 1 diabetes remain inconsistent, and ICI-induced type 1 diabetes has not been completely characterized. Further accumulation of cases and evidence is required to elucidate the characteristics and mechanisms of ICI-induced type 1 diabetes.

ICI-induced diabetes is frequently comorbid with other irAEs, including thyroiditis, hypophysitis, dermatitis, colitis, and adrenal insufficiency [1,16], though the causative relationships between irAEs and ICI-induced diabetes are unknown. Our patient had a 20-year history of chronic thyroiditis (Hashimoto’s thyroiditis), and his thyroid function was almost normal during treatment with levothyroxine (50 mg/day) 22 days before the first nivolumab administration. However, he transiently developed hyperthyroidism during admission due to FD, and he then developed more severe hypothyroidism that required treatment with a higher dose of levothyroxine (150 mg/day) for recovery of thyroid function. Among endocrine irAEs, hypothyroidism has been reported to be the most common irAE [1], and it is preceded by transient thyrotoxicosis in approximately half of the cases [1,17]. Our case also showed that process. Kotwal et al. [16] evaluated the baseline characteristics in patients with PD-1 inhibitor-induced diabetes and reported that 17% of the patients with new-onset type 1 diabetes had a personal history of Hashimoto’s thyroiditis and 8.3% had a family history of Hashimoto’s thyroiditis. Most recently, Kamitani et al. reported that patients with preexisting diabetes or hypothyroidism exhibited a significantly increased risk of ICI-induced type 1 diabetes, using the Japanese administrative claims database comprising 11 million patients [3]. As mentioned above, our patient had a 20-year history of Hashimoto’s thyroiditis treated with levothyroxine and a nine-year history of impaired glucose tolerance at the stable level of HbA1c (6.0%-6.1%) without any medication before nivolumab treatment. Regarding the association of preexisting endocrine diseases with ICI-induced type 1 diabetes or the frequent comorbidity among irAEs, the specific mechanism remains unknown. As speculative mechanisms, one could speculate that cross-reactivity or molecular mimicry among the antigens to ICIs in the tumor, endocrine tissues and pancreatic beta cells might cause inappropriate activation of T cells and acute onset of irAEs in target endocrine tissues which are possibly more vulnerable to ICIs-induced autoimmune activation [18], although specific antigens have not been identified or reported. In addition, preexisting smoldering inflammation that preceded ICIs but was unleashed by treatment could play a role in irAEs [1].

In most reported cases of ICI-induced diabetes, ICI treatment was continued when glucose levels were stabilized. The American Society of Clinical Oncology and National Comprehensive Cancer Network guidelines recommend withholding therapy until glucose control is achieved [19], although it has not been determined whether ICI treatment should be continued once glycemic control has been attained. Because most of the pancreatic beta cells have already been eliminated in cases of ICI-induced diabetes [20], treatment with multiple insulin injections has to be ongoing while continuing ICI treatment.

A limitation of our case was that we did not measure plasma glucose levels just before and after initial nivolumab treatment between days -1 and 6 during the first hospitalization, and we cannot completely exclude the possibility that our case developed type 1 diabetes before the initial nivolumab treatment, although the possibility is considered unlikely due to the lack of any symptoms and the stable laboratory data during the hospitalization between days -1 and 6. If we had measured the plasma glucose during the first hospitalization, we could have made a conclusion, and we might have found even earlier onset of FD and reported the time course of glucose elevation just after the initial treatment.

## Conclusions

We reported a case of FD that was diagnosed 10 days after the initiation of nivolumab therapy for gastric cancer. ICI-induced type 1 diabetes is a rare but potentially life-threatening irAE. Healthcare professionals should be aware of this serious irAE and repeat routine blood testing at short intervals from the first administration of ICI. Also, all patients, especially patients with a personal or family history of autoimmune endocrine diseases, diabetes, and even prediabetes, should be provided with information about symptoms of hyperglycemia and diabetic ketoacidosis before ICI treatment. Furthermore, novel biomarkers of susceptibility should be identified to better guide ICI treatment in the future.
