# Correction: Rapid Onset of Fulminant Type 1 Diabetes 10 Days After the First Dose of Nivolumab: A Case Report

**DOI:** 10.7759/cureus.c233

**Published:** 2025-07-29

**Authors:** Yoshinori Miyazaki, Tomomi Tateyama, Haruo Shimizu

**Affiliations:** 1 Diabetology, Muroran City General Hospital, Muroran, JPN; 2 Gastroenterology, Muroran City General Hospital, Muroran, JPN

This article has been corrected in paragraph 1 in the Case Presentation and paragraph 6 of the Discussion to reflect that the hospitalization days mentioned were days -1 and 6.

In the header row of Table 1, Day 33, Day 22, and Day 1 have been corrected to Day -33, Day -22, and Day -1.

